# Photothermal-Driven Liquid Crystal Elastomers: Materials, Alignment and Applications

**DOI:** 10.3390/molecules27144330

**Published:** 2022-07-06

**Authors:** Wei Zhang, Yifei Nan, Zongxuan Wu, Yajing Shen, Dan Luo

**Affiliations:** 1 Department of Electrical and Electronic Engineering, Southern University of Science and Technology, Shenzhen 518055, China; 12150028@mail.sustech.edu.cn (W.Z.); 11711413@mail.sustech.edu.cn (Y.N.); 12032222@mail.sustech.edu.cn (Z.W.); 2 Department of Biomedical Engineering, City University of Hong Kong, Kowloon, Hong Kong, China; yajishen@cityu.edu.hk

**Keywords:** liquid crystal elastomer, smart material, soft robot, actuator

## Abstract

Liquid crystal elastomers (LCEs) are programmable deformable materials that can respond to physical fields such as light, heat, and electricity. Photothermal-driven LCE has the advantages of accuracy and remote control and avoids the requirement of high photon energy for photochemistry. In this review, we discuss recent advances in photothermal LCE materials and investigate methods for mechanical alignment, external field alignment, and surface-induced alignment. Advances in the synthesis and orientation of LCEs have enabled liquid crystal elastomers to meet applications in optics, robotics, and more. The review concludes with a discussion of current challenges and research opportunities.

## 1. Introduction

Responsiveness to stimuli is the most attractive property of smart materials. More than half century ago, Pierre-Gilles de Gennes forecasted that liquid crystal elastomers (LCEs) exhibit strong stimulus-responsive properties [1]. Later, researchers found that LCEs obviously deform with various kinds of stimulation (such as light, heat, electricity, etc.), which was accompanied by a decrease in the order parameter [2,3,4,5,6]. Photo-responsive LCEs are attractive because properties of light, such as wavelength, intensity, and polarization direction, are easily modulated [7,8]. Photo-responsive LCEs are mainly based on two mechanisms, photochemical and photothermal. However, the former utilizes azobenzene materials that requires drive-light with high photon energy, which is always harmful to humans [9,10]. Therefore, increasing attention has focused on photothermal-driven LCEs.

The light-responsive properties of LCEs are based on material chemistry, processing, and alignment. LCEs are typically thermotropic liquid crystals (LCs), and when LCEs are heated to a temperature beyond the phase transition temperature, the liquid crystal mesogens lose their previous alignment and become randomly oriented, which results in macroscopic deformation [2]. To achieve photo-induced temperature changes, photothermal materials were added into LCEs. LCEs always contract along the director, and based on this, complex deformation can be achieved by controlling the alignment of LCEs [3].

This review explores the latest advances in photothermal-driven LCE materials and processing of LCE and LCE-based composite material. Firstly, the synthesis and basic properties of the LCEs will be introduced, and then several methods of LCE alignment (mechanical alignment, external field-induced alignment, surface-induced alignment) will be discussed, which can achieve alignments from simple to complex. Next, the applications of photothermally driven LCEs, ranging from optics, robotics, and actuators, will be demonstrated. At last, this review will conclude with a discussion of issues that need resolving and potential applications.

## 2. LCE and LCE-Based Composite Material

### 2.1. LCE

**Figure 1:**
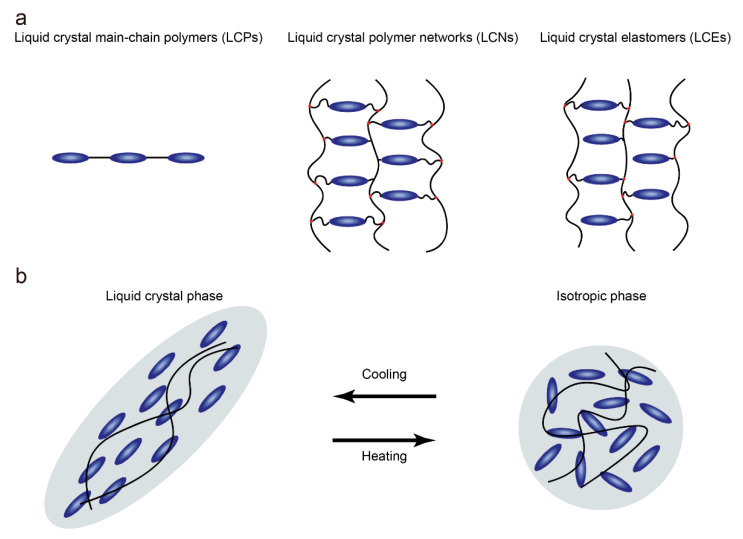
(**a**) Schematic of liquid crystal polymers, liquid crystal polymer networks and liquid crystal elastomers; (**b**) Schematic illustration of the contraction along the alignment of LCEs when heated beyond T_NI_.

Since Finkelmann et al. first reported liquid crystal elastomers in 1981, LCEs have under-gone tremendous development in both material chemistry and processing, becoming a class of materials with special properties [11]. Unlike liquid crystal polymers that are not crosslinked and liquid crystal networks that are highly crosslinked, low-crosslinked liquid crystal polymer networks (Figure 1a) with anisotropic polymer chains are known as LCEs [4]. Due to the low degree of crosslinking, anisotropic polymer chains can temporarily become isotropic when heated beyond the nematic-to-isotropic phase transition temperature (T_NI_), resulting in contraction along the alignment (Figure 1b). At the same time, the degree of order in the LCEs is reduced with the loss of anisotropic chain conformation. The presence of the polymer network ensures that the LCE can obtain the original orientation when the temperature is lower than T_NI_. This determines that the LCE undergoes reversible anisotropic deformation when the temperature changes. In fact, different application scenarios require LCEs that possess different orientations and physical properties, which are affected by the synthesis and alignment of LCEs. In general, the synthetic routes can be summarized into two groups: two-step crosslinking and one-step crosslinking. For the two-step cross-linking route, the LCE can be mechanically oriented because of the presence of a partially cross-linked polymer network. In the one-step crosslinking route, low molecular weight monomers are directly polymerized to form LCEs. Due to the low viscosity of the precursors, the LCEs prepared by one-step cross-linking are suitable for surface-induced orientation.

#### 2.1.1. Two-Step Cross-Linking

**Figure 2:**
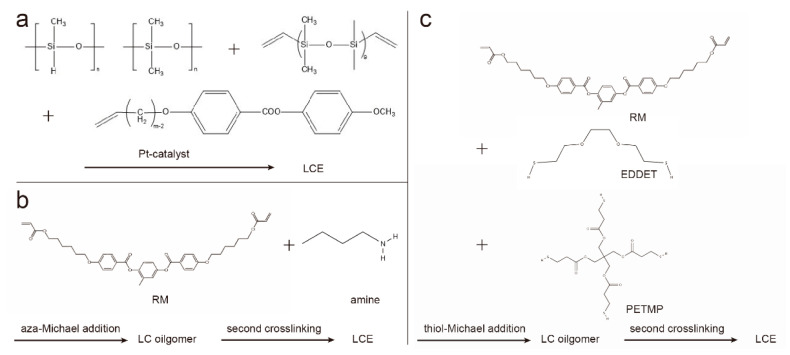
Synthetic route for two-step cross-linked LCE chemistry. (**a**) Two-step crosslinking by hydrosilylation; (**b**) aza-Michael addition between diacrylate-based RMs and amines; (**c**) thiol-Michael addition of diacrylate-based RMs and thiols.

The two-step crosslinking method proceeds in two steps, the first step forming a weakly crosslinked, partially reacted polymer network, whose shape and orientation can be easily changed. Through mechanical stretching and other methods, monodomain LCEs with the desired shape can be obtained, and the shape and orientation are fixed by the second-step reaction. A typical example of two-step cross-linking was presented by Finkelmann et al., which was based on liquid crystal polysiloxanes [11]. In the presence of a platinum catalyst, linear polysiloxane chains are mixed and reacted with vinyl liquid crystal monomers and multifunctional vinyl crosslinkers (Figure 2a). Taking advantage of the difference in the reaction rates of the two different cross-linking agents, the first-step reaction produces weakly cross-linked polymers, and aligns the polymer chains by mechanical stretching. Under the condition of weight loading, the second part of the polymerization reaction forms a strong cross-linked polymer network and permanently maintains a uniform director orientation.

The two-step hydrosilylation reaction route relies on non-commercially available materials. Therefore, the preparation of LCEs based on the chain extension reaction of diacrylate-based reactive mesogens (RMs) is gaining popularity, including the aza-Michael addition method [3,12] and thiol-Michael addition method [13]. Because both of the above mentioned methods are based on purchasable liquid crystal monomers and other components, the initiation conditions of the two-step polymerization are orthogonal based on the chain extension reaction, enabling easy access and good controllability. For the aza-Michael addition (Figure 2b), a classic example is based on the chain extension of liquid crystal diacrylate monomers via aza-Michael addition with amines [3,14], where the low viscosity of the reactive precursor and the slow reaction rate can be used for surface alignment [3,15]. The thiol-Michael addition reaction can be applied to chain extension to form oligomers (Figure 2c). After the alignment of oligomers, the orientation of the mesogens is then fixed by photopolymerization [16,17].

#### 2.1.2. One-Step Cross-Linking

Low molar mass liquid crystals have been studied for many years and are frequently used in daily life due to optical anisotropy and electrical tunability. Alignment techniques for low molar mass liquid crystals are well developed [18,19], which can also be used to align low-viscosity precursors of LCEs. Compared with the two-step cross-linking method, the low molar mass liquid crystal monomers are directly polymerized to form LCE in the one-step cross-linking method without the process of chain growth and partial cross-linking, which results in better maintenance of orientation. It is worth noting that this alignment technique is usually based on a treated surface, which possesses strong programmability, but the anchoring force weakens with distance. Due to the limitation of the anchoring force, LCEs fabricated by this alignment method are thin, and the LCE precursor is required to have a low viscosity. In the one-step cross-linking method, the polymerizable liquid crystal monomer, cross-linking agent, and initiator are directly mixed. Since no pre-polymerization process is required and the viscosity of the precursor is low, alignment can be induced by a surface to achieve spatially complex orientation. The first example of a one-step cross-linked LCE was synthesized by free-radical polymerization of acrylates [2]. Thomsen et al. used acrylate-functionalized LC monomers for crosslinking to obtain side-chain LCEs. Orientation was based on surface-induced orientation, where the material was poured into glass cells coated with a rubbed polyvinyl alcohol film, then cooled from the isotropic phase (95 °C) to the nematic phase (85 °C) at −1 °C/min to obtain a good orientation. This method only takes a few minutes to obtain LCEs, which saves time compared to two-step crosslinking.

### 2.2. LCE-Based Composite Material

An indispensable part of photothermally driven LCEs materials are photothermal materials. Photothermal materials can absorb light of specific wavelengths, transform photon energy into heat energy, and transfer the heat to LCEs for driving. The introduction of some kinds of photothermal materials can enhance the mechanical properties of LCEs as well. The research on photothermal conversion materials ranges from inorganic to organic, including carbon-based materials, metal nanomaterials, and organic dyes.

#### 2.2.1. Carbon Based Material (CNT, Graphene, GO)

**Figure 3:**
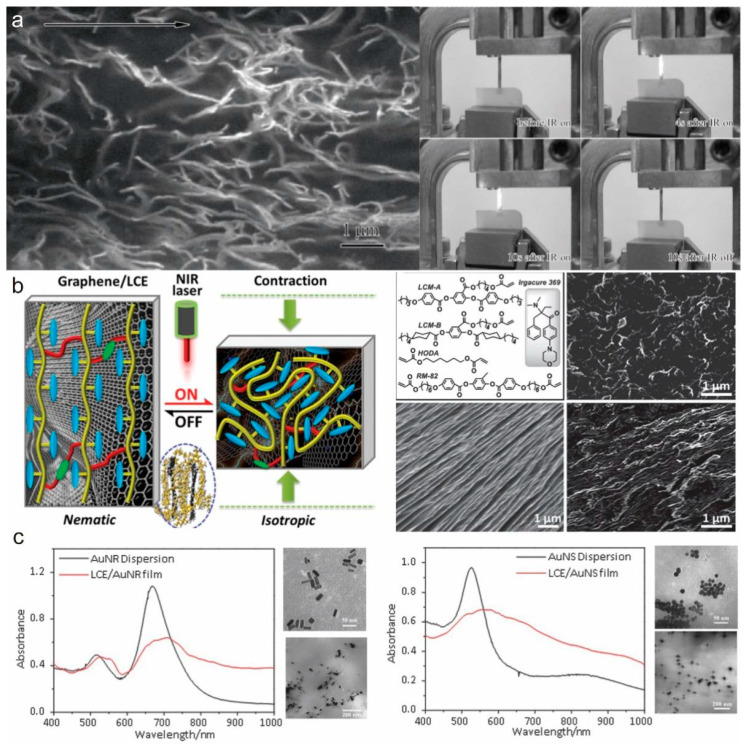
LCE composites. (**a**) CNT/LCE composite material (Reprinted with permission from Ref. [24]. Copyright 2008, John Wiley and Sons). (**b**) Graphene/LCE composite material (Reprinted with permission from Ref. [28]. Copyright 2015, John Wiley and Sons). (**c**) Metal nanomaterial/LCE composite material (Reprinted with permission from Ref. [30]. Copyright 2015, John Wiley and Sons).

Carbon-based materials are generally commercially available and generally have excellent thermal conductivity and mechanical properties. Carbon nanotubes (CNTs) are cylindrical tubes rolled from graphene sheets. The diameter of the cylinders is generally nanometers, and the lengths are in the range of several micrometers [20]. CNTs have the ability to convert luminous energy into heat energy and exhibit high photothermal conversion efficiency, so they are a commonly used photothermal conversion material in LCEs [21,22]. As early as 2003, Courty et al. [23] reported a composite of CNT and LCE. At that time, the mass ratio of CNTs was very low, less than 0.02 wt.%, due to the incompatibility of nanotubes and polymers, which made it difficult to distribute them uniformly in the LCE matrix at high concentrations. In order to obtain higher photothermal conversion efficiency and mechanical properties, increasing the CNT concentration has become an important proposition. In 2008, Yang et al. [24] changed the surface properties of carbon nanotubes without changing their intrinsic properties to achieve high dispersion of single-walled carbon nanotubes (SWCNTs) in an LCE matrix, and achieved reversible deformation driven by near-infrared light (Figure 3a). In 2010, Ji et al. [21] fabricated a pyrene-terminated LCE to facilitate the dispersion of carbon nanotubes and the LCE was then stretched to induce CNT alignment with the orientation of the LCE so that well-dispersed and aligned CNTs were obtained. Based on the excellent photo-mechanical properties of LCE-CNT composites, they have potential for applications in various fields. For example, artificial light-driven Braille displays have been reported [25]. Benefiting from the strong photothermal conversion efficiency of CNTs, a sun-driven sunflower-like actuator was fabricated, which can effectively improve the working efficiency of solar panels [26]. LCE-CNT composites were also applied to fabricate light-weight soft robots to realize multi-modal movements of crawling, squeezing, and jumping [27].

As one of the most popular materials, graphene has also been incorporated into LCE-based composites. Like CNTs, graphene exhibits good photothermal properties in the near-infrared region. Yang et al. [28] reported that by arranging graphene sheets in LCEs, the graphene sheets can act as nanoheaters and trigger the phase transition of LCEs, thereby achieving a light-driven macroscopic deformation of up to 35.7% (Figure 3b). Graphene has a tendency to self-aggregate, resulting in inhomogeneity of the composite. In contrast, graphene oxide (GO) contains oxygen-containing groups and carbonyl and carboxyl groups at the edges, so it is attractive for easy modification and dissolution. Li et al. [29] fabricated GO-LCE nanocomposite films that exhibited robust photo thermomechanical responses, reaching 33% uniaxial shrinkage and about 50% increase in payload actuation.

#### 2.2.2. Metal Nanomaterial (AuNP, AuNR)

Photothermal metal nanomaterials, such as gold and silver, produce strong absorption when the incident photon frequency matches the overall vibrational frequency of the metal nanoparticle, generating a large amount of heat. This is a phenomenon produced by localized surface plasmon resonance (LSPR). The powerful photothermal effect provides a new approach for photothermally driven LCE. In addition to the photothermal effect, Montazami et al. [31] found that embedding gold nanoparticles (AuNPs) into LCE could improve the thermal conductivity, Young’s modulus, and response speed of LCE. In this study, the response speed could be improved by more than 100%. Sun et al. [32] infiltrated micrometer-sized LCE cylindrical actuators with gold nanocrystals about 2 nm in diameter. The spatial translation, alignment, and rotation of the cylindrical microactuator were achieved by optical tweezers, and the bending deformation of the cylindrical microactuator was excited by a focused near-infrared (NIR) laser beam. Liu et al. [30] explored photothermal-driven LCE under visible light irradiation by dispersing gold nanospheres (AuNS) and gold nanorods (AuNR) directly in the LCE monomer (Figure 3c). The LCE/AuNS and LCE/AuNR composites were accessed by UV curing, achieving a driving strain of 30% under 635 nm laser irradiation. Xu et al. [33] fabricated gold nanoparticles/LCE nanocomposites using 5 nm gold nanoparticles. The absorption band of the gold nanoparticles was about 475 nm to nearly 625 nm, occupying a major part of the sunlight spectrum, resulting in a photothermal-driven LCE that can be driven by sunlight. However, gold nanoparticles also have the problem of agglomeration. Wójcik et al. [34] used nanoparticles as a cross-linking agent for LCE materials to realize uniform dispersion in the LCE matrix and ensure good orientation of the LCE.

#### 2.2.3. Organic Material (Organic Dye, PDA)

Compared with carbon-based composites and metal nanoparticles, organic materials are more likely to interact with LCE. Polydopamine (PDA) has attracted increasing attention because of its outstanding photostability and powerful photothermal effect in the near-infrared region. Tian et al. [35] used PDA to coat LCE films to fabricate optically responsive LCE films. By cleverly utilizing the property of PDA that can work in atmospheric and water environments at the same time, a bionic fish was manufactured that could swim on the water surface with NIR exposure. Lan et al. [36] made use of the rewritable properties of polydopamine coating to fabricate an LCE oscillator selectively coated with PDA. The powerful photothermal conversion ability of PDA allowed the LCE to be driven by sunlight, realizing the function of converting solar energy into electrical energy. Besides PDA, organic dyes such as disperse red have been widely used in LCE photothermal actuators and soft robots [14,15,17,27,37,38].

## 3. Alignment

To realize the controllable macroscopic deformation of LCE, the orientation design of the liquid crystal molecules must be carried out. Thus, various methods have been explored and applied to determine the orientation of the mesogens. Generally, existing methods for LC molecule alignment can be divided into three categories: mechanical stress alignment, external field alignment, and surface effect alignment.

### 3.1. Mechanical Alignment

The application of external stress can cause the long axis of the mesogens to shift either perpendicular or parallel to the direction of the force. Therefore, several types of stress are introduced into the preparation of monodomain LCE. For example, tensile stress, compressive stress, and shear stress are commonly applied to align LCE.

**Figure 4:**
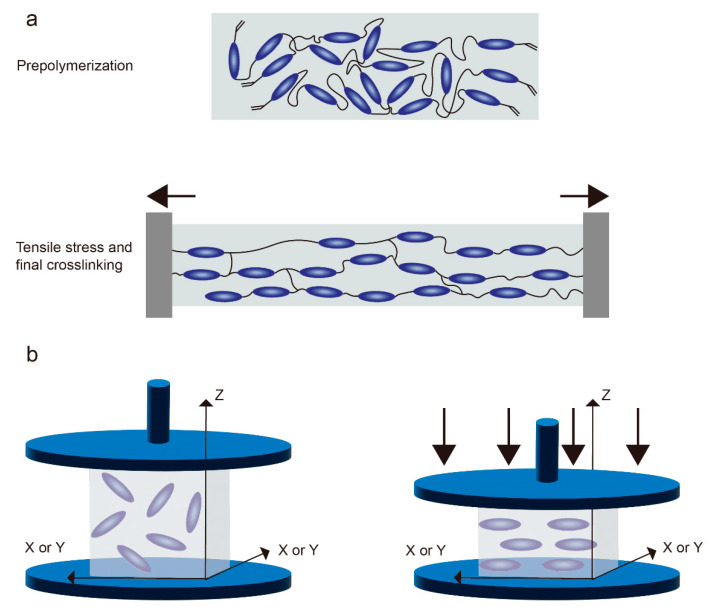
(**a**) Schematic diagram of LCE alignment by tensile stress. Mesogens will be aligned parallel to the direction of tensile stress. (**b**) Schematic diagram of LCE alignment by compressive stress. LC molecules will be oriented vertically according to the direction of the compressive stress.

Küpfer and Finkelmann [39] demonstrated that tensile stress could be used to oriented LCE for the preparation of unidirectional liquid crystal polysiloxanes. This method, also known as the Finkelmann method, is widely used because of its simple operation and excellent orientation effect. The LCE prepared by this method undergo two-step crosslinking [40]: the liquid crystal is pre-polymerized first, and then a certain pulling force is applied in the desired orientation direction (Figure 4a) and fixed by the second-step reaction, which has been mentioned above. In addition to the Finkelmann method, Jin et al. [41] designed another way to orientate LCE with a stretching force. They added a certain proportion of solvent to the liquid crystal precursor to prepare a fully cross-linked isotropic liquid crystal oil gel; once the solvent evaporates, the liquid crystal oil gels will be oriented according to the direction of stress applied to it.

The process of preparing LCE by compressive stress is similar to that of tensile stress (Figure 4b), but the direction of the mesogenic elements of LCE prepared by this method will be perpendicular to the direction of compressive stress [42,43].

**Figure 5:**
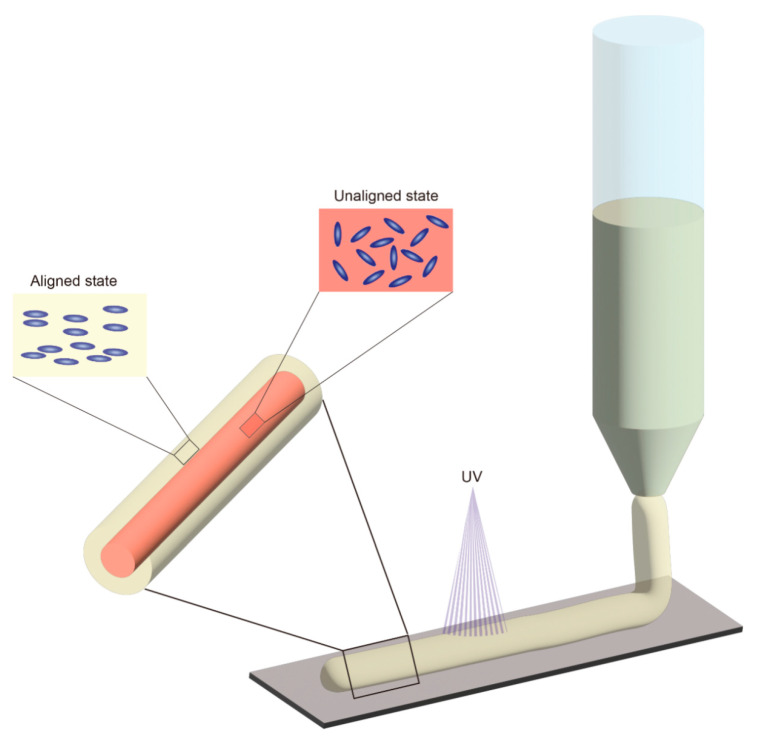
Schematic of direct ink writing (DIW) using shear stress aligned LCE. Mesogens will be aligned parallel to the direction of shear stress.

Extrusion orientation of liquid crystal precursors and thermoplastic liquid crystals by shear stress is also a very promising approach, which is well suited for incorporating LCE into 3D printing as a fourth dimension. A typical implementation of this method is direct ink writing (DIW). In DIW (Figure 5), the mixture of prepolymer and photoinitiator is heated to a temperature slightly beyond the T_NI_ then extruded from a nozzle and oriented by shear stress in the process, so that the orientation of the mesogens is parallel to the extrusion direction [44]. The layer-by-layer (row-by-row) fabrication of LCE three-dimensional structures can be conveniently achieved through the movement of the printing nozzles. After the mesogens are extruded, their outer regions are rapidly cooled and polymerized so their orientations are retained, while the inner regions gradually return to a multi-domain state. Therefore, the outer structure has a consistent orientation that is essential for stimulating deformation of an LCE fabricated by DIW. By changing the key parameters of printing (temperature, inner diameter of the nozzle, etc.), the volume ratio of the monodomain outer layer to the multidomain inner layer can be changed, thus changing the thermomechanical properties of the fibers [45].

### 3.2. External Field-Induced Alignment

Due to the special molecular structure of liquid crystal, its physical constants (such as refractive index, dielectric constant, diamagnetic elastic modulus, etc.) are typically anisotropic [46,47]. Liquid crystal molecules are endowed by this property with the ability to respond to stimuli from electric and magnetic fields. The responsiveness of liquid crystals to an external field is commonly used in the display field to control the direction of the liquid crystal and modulate light by changing the applied field [48]. This ability is now also being introduced for alignment and actuation of LCEs. External field-induced alignment is attractive because it can affect liquid crystal alignment in a non-contact way, which is accomplished by programming the field strength and direction.

#### 3.2.1. Electric Field

**Figure 6:**
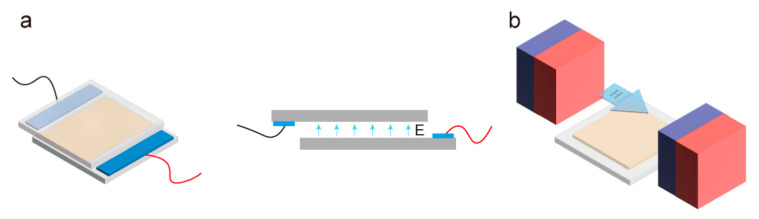
(**a**) Schematic representation of LCE alignment by electric field. (**b**) Schematic representation of LCE alignment by magnetic field.

Mesogens will deflect when in an appropriate electric field. The dielectric anisotropy of the LCE affects the deflection direction, and the electric field intensity affects the deflection degree [49]. When the dielectric anisotropy is positive, the dielectric constant of the liquid crystal along the long axis $\varepsilon_{∥}$ is greater than the dielectric constant along the short axis $\varepsilon_{⊥}$ [50] and the liquid crystal will align along the direction of the applied electric field (Figure 6a) [51]. For liquid crystals with negative dielectric anisotropy, such as most diacrylate liquid crystal monomers [52,53], the monomer will be deflected in a direction perpendicular to the external electric field. The strength of the electric field determines the degree of deflection of the mesogens. When the LCE is oriented by an applied electric field, the electric field strength of its aligned region must be higher than the threshold field strength $E_{c}$, which is given by

(1) $$E_{c}=\frac{\pi }{d}\sqrt{\frac{K_{i}}{\varepsilon_{0}\Delta \varepsilon }},$$

where $K_{i}$ is the Frank elastic coefficient, $\varepsilon_{0}$ is the permittivity of free space, $\Delta \varepsilon =\varepsilon_{∥}-\varepsilon_{⊥}$ is the anisotropy of the dielectric susceptibility, and d is the cell thickness [54,55].

You et al. [56] used striated indium–tin oxide (ITO) electrodes with different widths and distances on glass substrates to design cells that could align liquid crystal monomers by an electric field. Electrodes with pattern information made it possible to induce a periodic arrangement of the liquid crystal elastomer, which in turn changed the specific mechanical properties of the LCE such as surface friction coefficient, toughness, and ductility.

#### 3.2.2. Magnetic Field

The liquid crystal materials commonly used for the preparation of LCE basically have a benzene ring structure, and the strong diamagnetism [57] of this structure gives the liquid crystal molecules the possibility of being oriented by a magnetic field. For rod-shaped liquid crystal molecules, the magnetic susceptibility $\chi_{∥}$ parallel to the long axis of the monomers is not the same as the magnetic susceptibility $\chi_{⊥}$ vertical to the long axis of the mesogens (generally, $\chi_{∥},\;\chi_{⊥}<0,\;\left|\chi_{∥}\right|<\left|\chi_{⊥}\right|$). Similar to electric field-induced alignment, liquid crystal materials with positive (or negative) anisotropic diamagnetism will tend to align their long axis parallel (or perpendicular) to the direction of the applied magnetic field (Figure 6b). To achieve effective orientation of the mesogens, the intensity of the applied external magnetic field needs to be greater than the threshold field strength $H_{c}$, which is given by

(2) $$H_{c}=\frac{\pi }{d}\sqrt{\frac{K_{i}}{\mu_{0}\chi_{\alpha }}},$$

where $K_{i}$ is the Frank elastic coefficient, $\mu_{0}$ is the free space permittivity, $\chi_{\alpha }$ is the diamagnetic susceptibility, and d is the cell thickness [55].

Li et al. [58] achieved the encoding and local deformation of region-specific molecular anisotropy by introducing a magnetic field during the fabrication of liquid crystal elastomers. They poured LC blends into poly(dimethylsiloxane) (PDMS) molds with the desired microstructure and heated the LC mixtures above T_NI_. This strategy decouples the anisotropy of orientation from the directionality of the structure at the material level, providing the opportunity for a programmable dynamic equilibrium. Compared to mechanical stress alignment and surface alignment, the use of magnetic field orientation can provide region-separable, uniform orientation for large thicknesses or precision construction [58,59,60,61].

### 3.3. Surface-Induced Alignment

The practical application of LCE places a demand on the complex and high-resolution spatial alignment of liquid crystal monomers, which can be achieved by surface-induced alignment. There are various methods for achieving surface-patterned orientation, such as local friction [62], photolithographic patterning [63], and optical alignment, etc. The common strategy of these techniques is to create surfaces that induce the alignment of liquid crystal monomers.

#### 3.3.1. Alignment Layer

**Figure 7:**
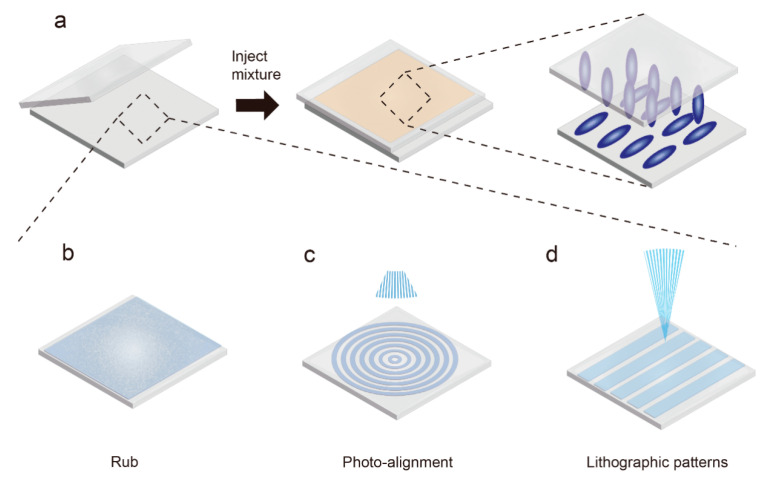
Schematic diagram of making an oriented substrate and surface-induced orientation of LCE. (**a**) Liquid crystal molecules aligned by layer; (**b**) fabrication of oriented substrates by friction; (**c**) production of oriented substrates by photosensitive materials and light sources with pattern information; (**d**) microchannels are formed on the substrate surface by lithography.

The alignment layer technology that is used extensively in the field of liquid crystal display can also be used in the alignment design of LCE [64]. There are two mechanisms contributing to the alignment (Figure 7a). On the one hand, the intermolecular force between the liquid crystal monomers and the alignment layer material is able to induce the alignment of the liquid crystal molecules. On the other hand, the orientation of the mesogens is further enhanced by creating tunnels in the alignment layer. A common method for creating microscale to nanoscale microchannels on the surface is to rub the alignment layer with a velvet cloth (Figure 7b). These microstructures will help aligning the long axis of the liquid crystal molecules with the microchannels. This phenomenon can be characterized by the anchoring energy density according to Berreman’s elastic deformation energy model for LCs aligned in one-dimensional grooves [63], which is given by

(3) $$\rho_{max}=\frac{\pi^{2}KA^{2}}{\lambda^{3}},$$

where K is the average elastic constant of the LCs, A and λ and are the amplitude and wavelength of the surface topography, respectively. $\rho_{max}$ is the anchoring strength of the LC on the one-dimensional channel, which characterizes the ability of the liquid crystal molecules to twist from other directions to the microchannel direction.

To solve the problem of generating a large amount of electrostatic charge dust and mechanical stress in the alignment layer during the rubbing process, a non-contact photo-alignment method can be used (Figure 7c). A common approach is spin-coating light-sensitive materials (such as azobenzene dyes [18,65], cinnamate [66], coumarin [67,68], etc.) on the substrate to align the liquid crystal molecules. Among the light-sensitive materials, azobenzene dyes are widely used due to their high photochemical reaction rate and strong alignment retention. The azobenzene dyes can be reversibly converted from trans isomers to cis isomers by irradiation with linearly polarized UV light, and then converted to trans isomers by heat or visible light irradiation. During this process, the long axis of the azobenzene dyes will tend to be perpendicular to the polarization direction of the UV light [69], and subsequently induce the alignment of the mesogens.

#### 3.3.2. Lithographic Patterns

The introduction of photolithography into the fabrication of alignment substrates can enable the orientation of liquid crystal molecules with arbitrary programmable (sub)micron resolution [63]. Microchannels can be selectively etched by photolithography on SU-8 photoresist with light irradiation, and then inverted to epoxy resin to produce alignment substrates with complex geometric patterns without the problems caused by charge and mechanical stress (Figure 7d).

In addition, another benefit of lithographic patterning used on oriented substrates for fabricating LCE is the ability to enhance the deformation complexity of the LCE. Aharoni et al. [70] demonstrated extraordinary pre-programming and control of LCE by combining the additive lithography pattern technique with inverse design.

## 4. Application

As a popular smart material, LCE have attracted widespread attention due to their stimulus responsiveness and programmability [4,32,69,71,72,73]. Photothermal-driven LCE is a competitive strategy because it can achieve remote control, wavelength selection, and accurate actuation. The powerful designability and controllability of photothermal-driven LCEs provide a variety of possibilities for applications. In this section, we will provide an overview of representative applications such as optical devices, soft robots, and actuators.

### 4.1. Optical Devices

**Figure 8:**
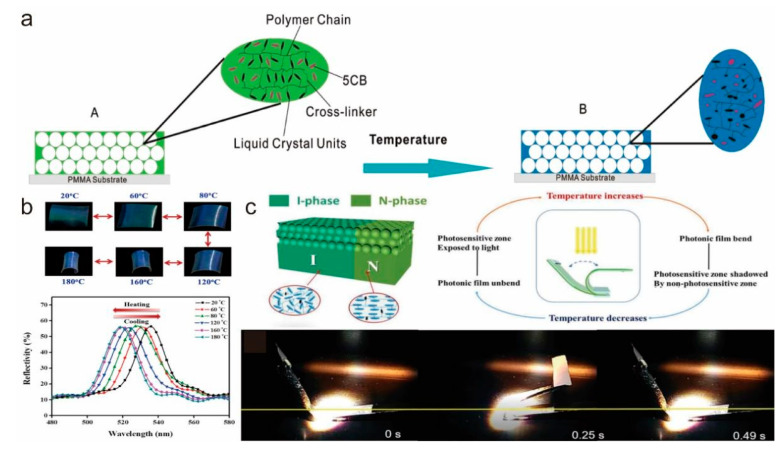
(**a**) Schematic diagram of the structure changing in an LCE-based inverse opal membrane with increasing temperature (Reprinted with permission from Ref. [82]. Copyright 2011, American Chemical Society). (**b**) Reversible driving behavior and reflectance spectrum shift of SiO_2_ opal PC/LCE composite films (Reprinted with permission from Ref. [80]. Copyright 2016, American Chemical Society). (**c**) Schematic diagram and demonstration of self-oscillation of two-segment photonic crystal thin films under visible light (Reprinted with permission from Ref. [83]. Copyright 2018, John Wiley and Sons).

The main characteristic of photothermal-driven LCE is that it can generate reversible large deformation under light stimulation, which can change lattice parameters and order degree in a photonic crystal structure. Therefore, combining LCE with photonic crystals (PCs) can create a responsive photonic crystal with a tunable photonic band gap (PBG) [74,75,76,77,78,79,80,81,82]. Wu et al. [82] created for the first time an LCE film with an inverse opal structure by infiltrating an opal structure formed by the self-assembly of silica spheres with a liquid crystal elastomer and then removing the spheres after polymerization (Figure 8a). The film could change the inverse opal structure under thermal stimulation, achieving a Bragg peak blue shift (more than 100 nm). Xing et al. [80] developed a SiO_2_ opal PC/LCE composite film actuator where macroscopic bending deformation and spectrum were controlled by a thermally induced nematic order change in the LCE molecules. Therefore, with an increase in temperature, the film showed obvious bending deformation and a PBG blue shift, which was reversible when the temperature decreased (Figure 8b). In addition to the inverse opal structure, cholesteric liquid crystal elastomers (CLCEs) can also form photonic crystals through self-assembly with thermally tunable reflection wavelengths [77]. These thermochromic optical devices have a prospect in light control. Although the above reports directly used thermal change to drive the LCEs, an LCE with a photonic crystal structure has the potential for photo-drive due to the development of photothermal conversion materials. Wei et al. [83] reported a photo-triggered dual-phase liquid crystal photonic actuator. Because of the photothermal effect of GO, the membrane has an inverse opal structure that can be reversibly bent when stimulated by light, and they employed selective excitation by photopatterned UV exposure in nematic/isotropic states to achieve complex shape changes and functions (Figure 8c). These results could provide inspiration for tunable optical devices and sensors.

### 4.2. Soft Robotics

Miniaturization and multiple degrees of freedom (DoF) are becoming the development trends in the field of smart robotics. A small structure with complex strain is difficult to achieve with traditional materials and fabrication methods, which can be overcome by the advantages of LCEs. LCEs can produce complex deformation due to patterned alignment when heated to phase transition temperature. By designing the alignment of LCE mesogens, soft robots with various functions have been developed, such as bionics, crawling, rolling, and swimming. In addition, the introduction of photothermal dopants enables the LCE robots to be driven accurately from a distance without being constrained by the energy source.

#### 4.2.1. Bionic Robots

**Figure 9:**
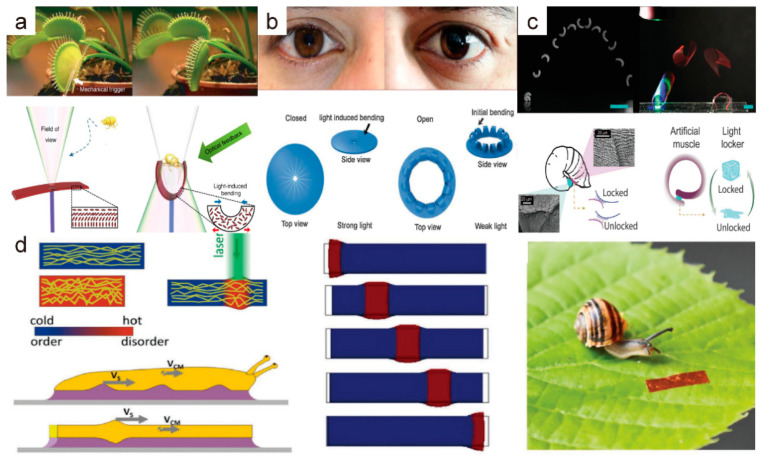
(**a**) Bionic light-powered artificial Venus flytrap robot and its working principle (Reprinted with permission from Ref. [5]. Copyright 2017, Springer Nature). (**b**) Light-driven LCE device simulating human iris structure and its negative feedback mechanism schematic (Reprinted with permission from Ref. [37]. Copyright 2017, John Wiley and Sons). (**c**) A light-controlled robot that can simulate the jumping of gall midge larvae and its energy storage and release mechanism (Reprinted with permission from Ref. [85]. Copyright 2021, John Wiley and Sons). (**d**) A snail-inspired LCE robot and its movement principle (Reprinted with permission from Ref. [84]. Copyright 2019, John Wiley and Sons).

Animals and plants in nature offer inspiration for designing robots. Sophisticated structures and intelligent systems enable creatures to perform their corresponding functions accurately. The stimuli-responsive properties make LCEs a suitable material for mimicking biological behavior. Wani et al. [5] designed and prepared a photothermal-driven LCE actuator that simulates a Venus flytrap and can identify and capture small objects (Figure 9a). When an object is located in the right place, light from the optical fibers connected to the center of the LCE film will be reflected onto the LCE Venus flytrap leaves. As a result, the LCE bends and traps the target due to photothermal-induced deformation. At the same time, the reflected light intensity is affected by the structure, material, and dimensions of the captured object, which provides information on the captured object for recognition by the device. When the light source is shut down, the deformation of the LCE recovers and the object is released. In addition to imitating plants, Zeng et al. [37] also used an LCE to design a structure inspired by iris tissue in the human eye that can adjust the power density of transmitted light by varying the degrees of deformation to adapt to the change in the power density of incident light. When the incident light intensity increases, the LCE curves inward and blocks the light, then returns to its initial state when the light intensity decreases (Figure 9b). This kind of negative feedback regulation is similar to the principle that adjusts pupil size to maintain a comfortable light intensity for the eyes. Rogóż et al. [84] designed a soft robot driven by a scanning laser beam and assisted by mucus (glycerin) based on studying and observing the movement mode of snails (Figure 9d). They combined the continuous deformation caused by a photothermal-induced phase change with the fluid properties of mucus to generate thrust and forward displacement, enabling the robot to achieve movement in horizontal, vertical, and obstacle conditions. Although this motion method was slow and environment dependent, it provides a unique vision for the study of LCE soft robots. Guo et al. [85] reported a latching and launching scheme for soft robots inspired by gall midges (Asphondylia). They used a photo-responsive LCE as an energy storage, storing elastic energy under blue light, and a liquid crystal adhesive as an energy latch. The adhesive produced a reversible photothermal crystal-liquid transition under the stimulation of green light, and then released the elastic energy instantaneously, enabling the soft robot to jump (Figure 9c). This brings new inspiration to the motion modes of bionic robots under light control.

#### 4.2.2. Crawling Soft Robots

**Figure 10:**
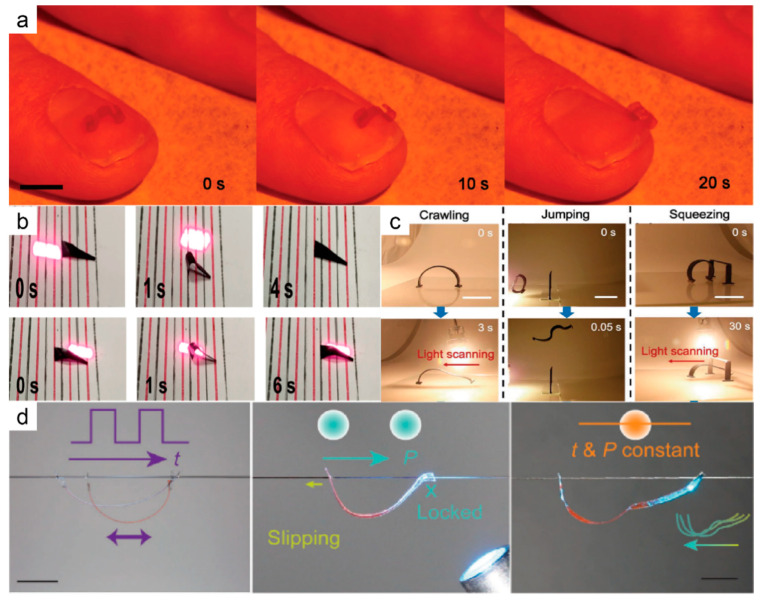
(**a**) Caterpillar robot crawling on a human fingernail under the irradiation of a 488 nm laser (Reprinted with permission from Ref. [87]. Copyright 2018, John Wiley and Sons). (**b**) Image of a trapezoidal LCE robot crawling forward and backward under near-infrared light (Reprinted with permission from Ref. [93]. Copyright 2022, American Chemical Society). (**c**) Multimodal locomotion of a soft robot powered by light (Reprinted with permission from Ref. [27]. Copyright 2019, John Wiley and Sons); (**d**) A robot moves along a hair in different patterns. One is friction control, which uses light to lock and slide between the legs and the other is a self-oscillating mode (Reprinted with permission from Ref. [89]. Copyright 2022, John Wiley and Sons).

Crawling is one of the simplest modes of movement. Photothermal-driven LCE soft robots can crawl in complex and extreme environments because of the softness of the body [7,27,86,87,88,89,90,91,92,93,94,95,96]. In addition, the robots can be made smaller because they do not need to carry a power source like traditional robots, as light can provide energy remotely. Anisotropic force is the basic requirement to realize crawling, and usually, this uneven force is achieved through special structural or surface designs. Zeng et al. [87] prepared a crawling robot based on a single-layered liquid crystal elastomer that can move on different kinds of surfaces under the stimulation of visible light. Since the movement is stimulated by low-intensity visible light, the soft robot has high safety and can be operated on human skin (Figure 10a), which is not possible with photochemical-driven LCE (because azo-LCE requires high photon energy). Yu et al. [93] constructed a soft robot from liquid crystal elastomers, phase change polymers, and carbon nanotubes that could be driven by infrared light. This composite material had good optical drive performance and could deform and recover fast. They also designed a soft robot with a trapezoidal structure (Figure 10b); because the two edges of the trapezoid had different contact areas, the soft robot could move in two directions on a straight line through the control of light. Ahn et al. [27] designed a multimodal soft robot based on composites of LCE and CNT. The robot could crawl, jump, and squeeze driven by a light bulb used in daily life (Figure 10c). The addition of CNT not only contributes to photothermal conversion, but also enhances the mechanical properties of materials, which provides support for expanding the potential application scenarios for photothermal-driven LCEs. Cheng et al. [89] reported a light-driven LCE film that could crawl along a hair (Figure 10d). The micro-structure of the hair’s surface provided different friction coefficients in different directions, allowing the soft robot to move along the hair in a single direction when stimulated by a manual scanning light beam. They also explored other patterns of motion on human hair, such as vertical crawling with light switchable friction and self-oscillating motion, providing more new ideas for soft robot control in restricted environments.

**Figure 11:**
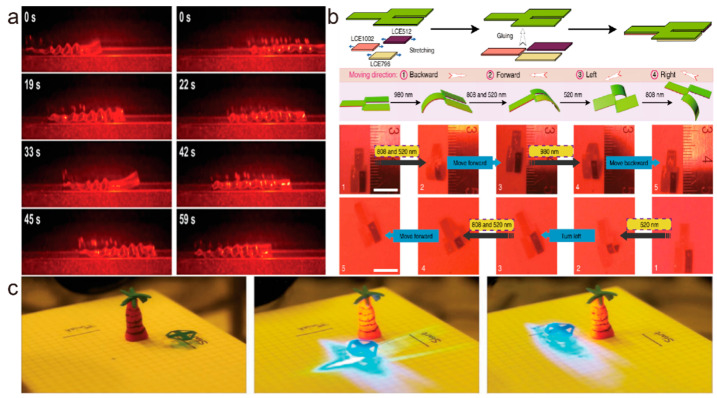
(**a**) A soft robot moving forward and backward under a continuous wave green laser (Reprinted with permission from Ref. [86]. Copyright 2016, John Wiley and Sons). (**b**) Schematic diagram of a multi-directional soft robot, and images of it crawling under three wavelengths of light (Reprinted with permission from Ref. [7]. Copyright 2019, Springer Nature). (**c**) A light-driven soft robot moving around (multi-directional) a palm tree (Reprinted with permission from Ref. [91]. Copyright 2020, John Wiley and Sons).

Multi-directional crawling has also been explored by researchers. Rogóż et al. [86] reported an LCE soft robot that could achieve one-dimensional bidirectional locomotion under the control of a scanning continuous wave green laser beam. The robot was made from a liquid crystal cell with alternating alignment layers that was able to create a traveling wave deformation under the photothermal effect. When the laser beam scanned along the robot, the robot moved in the direction of the scan, thus, by controlling the scanning path of the laser beam, they could make the soft robot move forward or backward (Figure 11a). This kind of driving mode endows LCE soft robots with better freedom of locomotion and reduces the demands of the working environment. Zuo et al. [7] prepared three kinds of LCE actuators that responded to different wavelengths of light and assembled them into a soft robot that could locomote in multiple directions. These LCE actuators were driven by 980 nm, 808 nm, and 520 nm lasers; thus, the robot could move forward and backward, and turn left and right by using muti-wavelength light (Figure 11b). This multi-wavelength modulation enhances the flexibility of LCE soft robot locomotion and make it possible to drive like a car. Cunha et al. [91] designed a multi-functional robot driven by blue light. The robot could move independently in four directions without turning (Figure 11c) and had the ability to carry and release objects. It had four symmetrically distributed legs and a set of arms. The initial state of these legs and arms was bent, so when they were illuminated by blue light, they straightened to achieve the stride or release. The structural design of this soft robot combined multi-directional movement and transportation functions together for the first time, which further improved locomotion mobility and opened the door for the practical application of crawling liquid crystal polymer soft robots.

#### 4.2.3. Rolling Soft Robots

**Figure 12:**
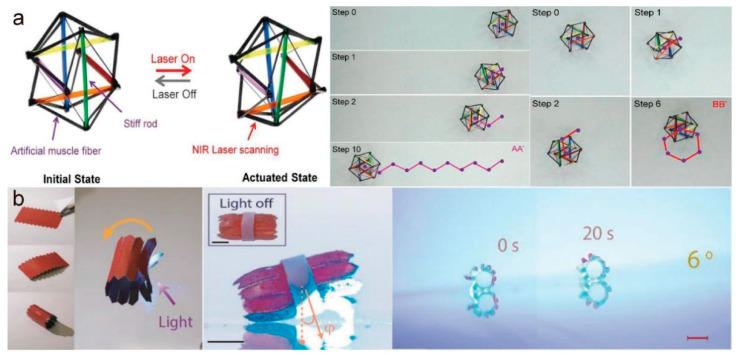
(**a**) Light-induced deformation schematic diagram of a tensegrity robot and demonstration of zigzag and gyro rolling of the robot under irradiation with a NIR laser (Reprinted with permission from Ref. [96]. Copyright 2019, John Wiley and Sons). (**b**) Design of a light-driven rolling robot, and images of the robot climbing a 6° hill (Reprinted with permission from Ref. [97]. Copyright 2020, John Wiley and Sons).

Rolling is a very common form of motion due to its low friction. Generally speaking, rolling has higher speed and lower complexity than most of motion modes, which is suitable for completing simple transportation work. Wang et al. [96] designed a photo-responsive tensegrity robot that was ultra-light and impact resistant with a high load capacity, and was able to accurately move in multiple directions in various terrain (Figure 12a). They used LCE-CNT composite fibers for the robot’s muscles and rigid rods as bones, forming an icosahedron. When one of the fibers was stimulated with near-infrared light, the robot’s center of gravity shifted, resulting in the whole structure rolling. The rolling robot could carry 7.5 times its own weight without affecting the photothermal-driven motion. In addition, the robot could successfully roll down from a height with the structure intact while carrying an egg and could roll on sand and gravel surfaces at a normal speed. Kirigami has received extensive attention in the field of soft robot research in the last few years. Cheng et al. [97] designed a kind of kirigami-based photothermal-driven rolling robot with a strong locomotion ability from a rolling gait. It generated intermittent 2D movement under the control of light and could roll up a 6° slope (Figure 12b). The combination of kirigami and photothermal-activated materials provides new inspiration for exploring the design of photothermal-driven soft robots.

#### 4.2.4. Swimming Soft Robots

**Figure 13:**
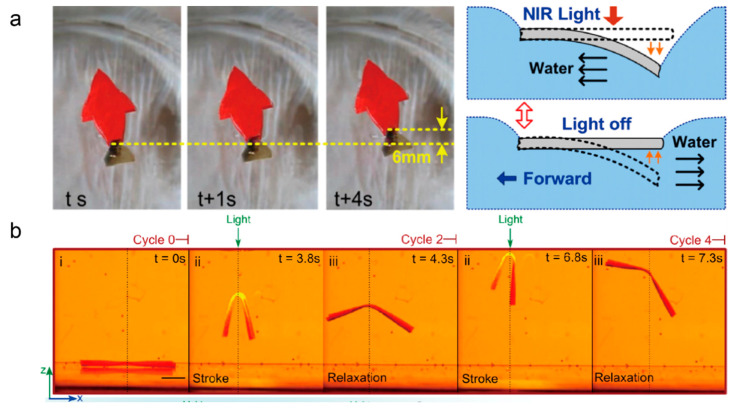
(**a**) Light-driven swimming of a soft robot based on an LCE film coated with PDA, and the schematic diagram of its actuation principle (Reprinted with permission from Ref. [35]. Copyright 2018, American Chemical Society). (**b**) Vertical locomotion of a soft robot underwater reported by Hamed Shahsavan et al. (Reprinted with permission from Ref. [99]. Copyright 2020,National Academy of Sciences).

As a variety of soft robots that can move on land are designed, researchers have gradually turned their attention to swimming soft robots that can shift freely in water. The cordless drive and material stability provide the possibility of working in extreme underwater conditions, such as deep sea or corrosive solutions. Palagi et al. [98] demonstrated a miniature LCE swimming soft robot that can perform complex locomotion driven by structured light. The scanning of structured light causes the micro-robot to periodically deform locally, thus generating self-propulsion. They created a long cylinder and a flat disk LCE soft robot capable of translating and rotating, respectively, in a liquid environment. This kind of structured light field use enables simple robots to achieve complex functions, which is an important step in the research of micro-robot control methods. Generally speaking, the low driving force generated by photothermal-driven LCE and the rapid heat dissipation in water have always been important factors limiting their application in water. Tian et al. [35] prepared a polydopamine (PDA) coated LCE and designed a soft swimming robot that could be driven by near-infrared light. The PDA-coated LCE film was used as the swimmer’s tail fin. Due to the strong cooling capacity of water, the tail fin bent relatively slowly under NIR light and recovered quickly after the light was turned off. The elastic force of recovery pushed the water back, causing the swimmer to move forward (Figure 13a). Restricted by the absorption of heat of LCE in the water environment, there are few studies on LCE swimming robots. However, with the development of photothermal materials, there will be more and more research related to LCE swimming robots. Shahsavan et al. [99] demonstrated a miniature underwater soft robot, which had low stiffness, density, and T_NI_, and could swim under the irradiation of light (Figure 13b). When exposed to light, the robot bent and produced a downward stroke, while its density also decreased, thereby increasing its buoyancy. A soft robot made of this material successfully achieved vertical movement in water, which is an important step for the underwater application of LCE soft robots.

### 4.3. Actuator

In recent years, many kinds of photothermal-driven LCE actuators have been reported. Among them, an oscillator can generate periodic actuation under constant light stimulation, and an artificial muscle can be excited by light to generate large force. Therefore, in this section, we will examine these two interesting actuators with potential applications.

#### 4.3.1. Oscillator

**Figure 14:**
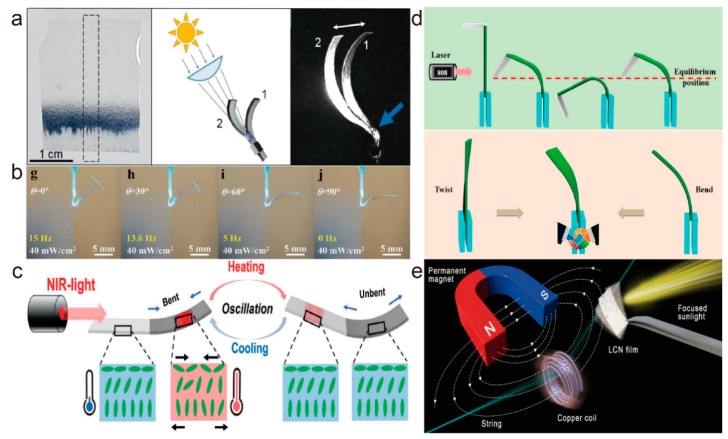
(**a**) Schematic diagram of an indigo dye-doped LCE film self-oscillating in sunlight (Reprinted with permission from Ref. [100]. Copyright 2017, John Wiley and Sons). (**b**) Oscillation images of dichromatic dye-doped LCE films at different θ (0°, 30°, 60°, and 90°) and a fixed laser power density (40 mW cm^−2^) (Reprinted with permission from Ref. [8]. Copyright 2020, John Wiley and Sons). (**c**) Schematic diagram of self-masking oscillation of a PDA-coated liquid crystal polymer film (Reprinted with permission from Ref. [36]. Copyright 2020, John Wiley and Sons). (**d**) The process of light-driven motion and deformation of a composites film under the irradiation of a NIR laser (Reprinted with permission from Ref. [101]. Copyright 2022, American Chemical Society). (**e**) Schematic diagram of a miniature solar generator (Reprinted with permission from Ref. [36]. Copyright 2020, John Wiley and Sons).

The self-oscillating behavior of actuators has attracted great attention in last few years because of the periodic motion generated by a constant stimulus. Although most reports have been concerned with azobenzene-based liquid crystal polymers, self-oscillators based on photothermal effects have also been demonstrated. Broer et al. [100] developed an LCE film doped with light stabilizers that can be driven by sunlight to oscillate based on a self-shading effect with the addition of indigo dye (Figure 14a). This research is the first report of a photothermal-driven LCE oscillator driven by sunlight. Li et al. [8] prepared a linearly polarization-dependent photothermal-driven soft actuator based on dichroic dye-doped LCEs, and further demonstrated its properties as an oscillator. The frequency of this oscillator was determined by the polarization of light. When laser power is constant, the frequency of oscillation increases as the deflection angle decreases (Figure 14b). At a laser power of 43 mW cm^−2^ and θ = 0°, this oscillator achieved a maximum frequency of 16.7 Hz. Lan et al. [36] designed and fabricated a novel photothermal-driven oscillator based on a PDA-coated liquid crystal polymer (Figure 14c). The PDA coating has good light absorption and stability and can enhance the frequency of the oscillator. Compared to other methods, PDA coatings are easier to remove, allowing for recoating and patterning. The same team also created a simple solar generator with a PDA-coated oscillator (Figure 14e). By slapping a copper coil to cut the magnetic induction line, it succeeded in generating a tiny voltage. In another example, a graphene Oxide (GO)/liquid crystal network actuator with multiple modes of oscillation was reported by Wang et al. [101]. This actuator can produce an oscillation mode coupled with bending and torsional oscillations under near-infrared light, providing another possibility for the oscillating behavior of a light-driven self-oscillator (Figure 14d).

#### 4.3.2. Artificial Muscles

Artificial muscles that can assist or replace biological tissue are becoming a research hotspot. As early as 1975, Pierre-Gilles de Gennes proposed that LCE, a material capable of producing large stresses and strains under a variety of external stimuli, as one of the most suitable candidates for the manufacture of artificial muscles [102]. At present, many actuation modes have been constructed to actuate LCE artificial muscles, such as direct-heated [103,104,105], electrothermal [106,107] and photothermal [108,109,110].

**Figure 15:**
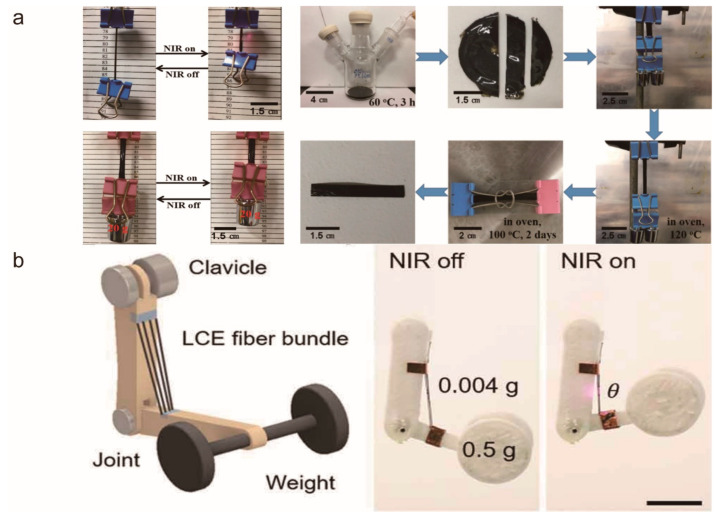
(**a**) Images of an LCE film lifting up a binder clip and load under NIR light illumination (left) and the preparation process of the film (right) (Reprinted with permission from Ref. [107]. Copyright 2017, American Chemical Society). (**b**) Schematic and image of an LCE fiber arm lifting a heavy object (Reprinted with permission from Ref. [110]. Copyright 2021, the American Association for the Advancement of Science).

Compared with other driving methods, photothermal-driving is safer because it can use visible light or NIR light that is harmless to the human body, and it is non-electric. Liu et al. [107] developed a NIR dye-doped liquid crystal elastomer actuator based on the two-step crosslinking method. The actuator can respond quickly to NIR light and lift up to 5680-times its own weight (Figure 15a). Its excellent mechanical properties demonstrate its potential for artificial muscles. Ferrantini et al. [108] prepared and characterized a biocompatible light-responsive acrylate-based LCE. The material can be activated at very low light intensity and the contraction kinetics do not change for nearly a month. In addition, by mounting LCE strips in parallel with a mouse cardiac trabecula, they showed that it was effective in helping the heart contract, developing a contractile force three times higher than that of human myocardium. Zhang et al. [109] synthesized a photothermal-driven CNT/LCE elastomer. This material can be driven by light in the 200–1200 nm region, and has satisfactory mechanical toughness, drive performance, deformation amplitude, and stability. It can produce a maximum actuation stress of 1.66 MPa and a strain of 400%, which satisfies both the actuation stress (>0.35 MPa) and strain (>40%) requirements of artificial skeletal muscles. In addition, they further prepared a bionic hand with CNT/LCE actuators as tendons in which each actuator can be independently controlled with high precision by near-infrared laser irradiation. These studies on artificial muscles have played a powerful part in promoting the progress and application of photothermal-driven LCE materials in the medical field. He et al. [110] used electrospin technology to fabricate an LCE microfiber and enabled it with the response ability to near-infrared light by coating it with PDA. They also used this fiber to simulate the process of lifting a heavy object like the human arm (Figure 15b), demonstrating that it could perform very similarly to human muscle. The fast responsiveness and large strain amplitude of this microfiber actuator opens up new possibilities in the field of artificial muscles.

## 5. Conclusions

In conclusion, the mechanical and response properties of LCEs can be controlled by the synthesis method and material composition. A properly cross-linked liquid crystal polymer network is the key for a strong stimulus response. For photothermal-driven liquid crystal elastomers, appropriate photothermal materials can not only obtain good photothermal conversion efficiency, but also increase the mechanical properties of LCEs. New synthetic methods and composite materials can bring new applications and functions to LCEs.

The deformation behavior of LCEs is usually controlled by orientation, and mechanical orientation is the most common method used to fabricate uniaxially oriented LCEs. Other orientation methods, such as surface-induced orientation, allow LCEs to generate complex alignment in three-dimensional space for adapting to various applications in optical devices, soft robots, and actuators.

Advances in synthesis methods, material processing, and orientation technologies have greatly promoted the development of photothermal-driven LCEs. However, ultra-fast response speed and great strain are still challenges. Stronger mechanical properties enable LCE to crawl, swim, jump, and even fly, giving LCE soft robots powerful movement abilities and provide LCE actuators with a stronger load capacity and working speed. At present, the accurate deformation control of LCEs is still a problem, and it is believed that advanced control strategies [111,112,113,114] can solve this problem in the future, making LCEs competent for more precise work, such as surgical robots and micro/nano manipulation.

## Data Availability

Not applicable.
